# Clean Hospitals Day 2023 marks the global launch of a self-assessment tool

**DOI:** 10.1186/s13756-023-01315-y

**Published:** 2023-10-08

**Authors:** Alexandra Peters, Pierre Parneix, Didier Pittet

**Affiliations:** 1 Infection Control Programme, Faculty of Medicine, University of Geneva, 4 Rue Gabrielle-Perret-Gentil, 1211 Geneva 14, Switzerland; 2 Institute of Global Health, Faculty of Medicine, Geneva, Switzerland; 3 Nouvelle Aquitaine Healthcare-Associated Infection Control Centre, Bordeaux University Hospital, Bordeaux, France; 4 French Society for Hospital Hygiene, Brest, France

**Keywords:** Infection prevention and control, Infection control, Environmental hygiene, Self-assessment, Healthcare-associated infection, Multimodal strategy, Environmental cleaning

**Fig. 1:**
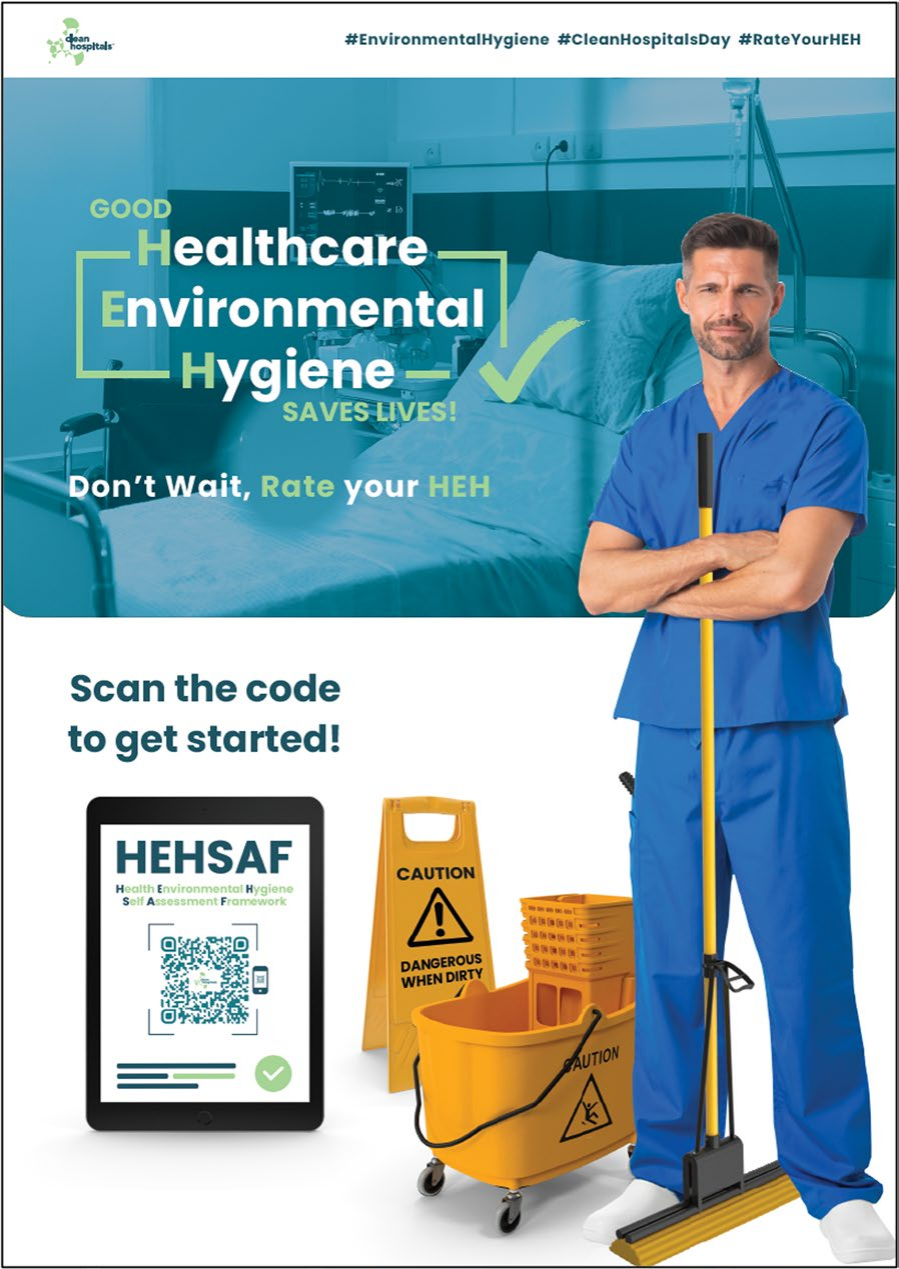
One of the promotional posters for Clean Hospitals Day, 2023

Healthcare environmental hygiene is increasingly recognized as critical in infection prevention and control (IPC). Much like its predecessor World Hand Hygiene Day, the objective of the Clean Hospitals Day, celebrated each year on October 20, is to raise awareness and foster engagement among healthcare facilities around the world. The newly published tool, the Healthcare Environmental Hygiene Self-Assessment Framework (HEHSAF), has been developed and internationally validated to help healthcare facilities to identify areas for improvement in their environmental hygiene programs, as well as benchmark this improvement over time. Healthcare facilities can download the full promotional toolkit and posters (Fig. 1) for Clean Hospitals Day 2023 from: www.CleanHospitals.com.

The launch of the HEHSAF tool in at least six languages (Chinese, Croatian, English, French, Portugese, Spanish and Turkish) marks the first time that a global snapshot of healthcare environmental hygiene programs is being attempted. The tool is geared towards IPC experts and environmental hygiene managers to help them identify areas for improvement in their facilities. It has been in development since 2018, and consists of a 96-question secure, online tool based on a multimodal improvement strategy. A pilot study using an earlier version of the tool was conducted by Clean Hospitals in 51 healthcare facilities in 35 countries [1]. It was then further developed with the help of an international expert group and validated internationally in seven additional countries.

The HEHSAF is published on the online platform REDCap, a secure web application for building and managing online surveys and databases. It is specifically geared to support online and offline data capture for research studies and operations. Available exclusively to institutions, REDCap is used by the most prestigious universities around the world. Currently over 6600 institutions in over 150 countries trust the security of the platform [2]. Although healthcare facilities who complete the HEHSAF will have access to their own detailed data, only anonymized and aggregated data will be shared. This is of utmost importance to ensure that healthcare facilities are comfortable being transparent when filling out the tool.

With global implementation, the HEHSAF will give institutions a roadmap for improving their environmental hygiene, support environmental services staff, increase the visibility of healthcare environmental hygiene on a global level, and save lives by reducing healthcare-associated infections. The HEHSAF and a full Clean Hospitals Day toolkit can be found on the Clean Hospitals website (www.CleanHospitals.com). The tool is also available at the following link: https://redcap.link/HEHSAF.

Let’s improve healthcare environmental hygiene together and save lives.
